# Intersectional Discrimination Is Associated with Housing Instability among Trans Women Living in the San Francisco Bay Area

**DOI:** 10.3390/ijerph16224521

**Published:** 2019-11-15

**Authors:** Theo Beltran, Amani M. Allen, Jess Lin, Caitlin Turner, Emily J. Ozer, Erin C. Wilson

**Affiliations:** 1 School of Public Health Division of Community Health Sciences and Epidemiology, University of California, Berkeley, CA 94720, USA; amaniallen@berkeley.edu (A.M.A.); eozer@berkeley.edu (E.J.O.); 2 San Francisco Department of Public Health, San Francisco, CA 94102 USA; jess.lin@sfdph.org (J.L.); caitlin.turner@sfdph.org (C.T.); erin.wilson@sfdph.org (E.C.W.)

**Keywords:** trans women, discrimination, housing

## Abstract

Trans women face numerous structural barriers to health due to discrimination. Housing instability is an important structural determinant of poor health outcomes among trans women. The purpose of this study was to determine if experiences of intersectional anti-trans and racial discrimination are associated with poor housing outcomes among trans women in the San Francisco Bay Area. A secondary analysis of baseline data from the Trans *National study (*n* = 629) at the San Francisco Department of Public Health (2016–2018) was conducted. Multivariable logistic regression was used to analyze the association between discrimination as an ordered categorical variable (zero, one to two, or three or more experiences) and housing status adjusting for age, years lived in the Bay Area, and gender identity. We found that the odds of housing instability increased by 1.25 for every categorical unit increase (1–2, or 3+) in reported experiences of intersectional (both anti-trans and racial) discrimination for trans women (95% CI = 1.01–1.54, *p*-value < 0.05). Intersectional anti-trans and racial discrimination is associated with increased housing instability among trans women, giving some insight that policies and programs are needed to identify and address racism and anti-trans stigma towards trans women. Efforts to address intersectional discrimination may positively impact housing stability, with potential for ancillary effects on increasing the health and wellness of trans women who face multiple disparities.

## 1. Introduction

Trans women have a higher likelihood of unmet basic needs than the general population, such as inadequate food and housing, which have in turn been associated with poor health outcomes [1,2,3]. A study in Los Angeles (*n* = 517) that analyzed the relationship between hormone use and structural inequities (income, housing, and health insurance) among trans women found that more than half of study participants were unstably housed, 22.4% were marginally housed, and 34.8% were homeless [4]. The National Center of Transgender Equality reports that one in five transgender people have experienced homelessness in their lives [5]. Trans women in San Francisco may be particularly impacted by housing instability due to the growing housing crisis locally [6]. The San Francisco Bay Area has one of the highest costs of living indexes in the United States compared to the U.S. average, and only 16% of housing in San Francisco is considered “affordable,” where rent amounts to 30% of one’s household income [7].

Housing instability is associated with poor health outcomes among trans people [3]. Unstable housing is associated with depression, higher risk for HIV acquisition, low antiretroviral therapy (ART) adherence, and viral suppression, and substance use for trans women [1,4,6,8,9,10,11,12,13,14]. Housing is directly related to health, as housing instability, having a low income, and having no health insurance are also factors associated with less utilization of health care services that could increase preventative behaviors and improve health outcomes [15]. Research in HIV also points to the impact of housing on health among trans women. For example, research on HIV care outcomes among trans women living with HIV in the San Francisco Bay Area found that housing instability was associated with poor HIV care outcomes [5]. Precursors to social determinants inequities are associated with low pre-exposure prophylaxis (PrEP) uptake among trans women compared to men who have sex with men (MSM) in San Francisco [16].

There are direct forms of discrimination that impact housing for trans women. For example, anti-trans discrimination has also been documented in shelters; the majority of trans women report experiencing homelessness at least once in their life, which may be linked to discrimination in shelters [17,18]. Studies focusing on trans women have also found that social structures, stigma, and rejection from family and friends may affect the ability to secure housing [19]. A study on lesbian, gay, bisexual, and transgender (LGBT) youth homelessness in New York City found that familial acceptance impacted housing status at an early age but called for more studies to focus on the risks leading to homelessness at the institutional level [20]. Trans women rejected by family and others who provide them shelter may be forced out of being housed because of their identity. Structural risks from discrimination may also lead to housing instability. Trans women experience disproportionate experiences of discrimination [21,22,23,24,25,26,27,28] leading to structural barriers that cause poverty, unemployment, limited access to health care, psychological distress, post-traumatic stress disorder (PTSD), and greater risk of experiencing violence [13,17,23,29]. Trans women may also be denied housing because their work may not be documented as a formal source of income, their housing history is limited, or they do not have an acceptable credit score.

In addition to anti-trans discrimination, many trans women experience interlocking forms of discrimination, or intersectional discrimination. Intersectionality was coined by Crenshaw and has been operationalized as the compounding effects of race, class, gender, ability, sexuality, and/or age on marginalization and health [30]. Overlap in more than one of these identities can contribute to inequities at the structural level (i.e., education and employment) [31,32]. Past research has shown that the burden of anti-trans discrimination is most felt by those with multiple marginalized identities within the trans women community, including trans women who are racial/ethnic minorities, and trans women experiencing homelessness [31,32,33,34,35]. Yet little research has demonstrated how intersectional racial and anti-trans discrimination impacts housing. While past literature has shown housing to be a predictive factor of risk and health, housing is rarely framed as an outcome, particularly for trans women.

The purpose of this analysis was to assess associations between anti-trans, racial, and intersectional discrimination and housing status of trans women living in the San Francisco Bay Area. Our research question is the following: “Are self-reported experiences of discrimination due to trans identity and/or being a racial/ethnic minority associated with housing instability among trans women?” Few studies in the San Francisco Bay Area have analyzed how lifetime experiences of anti-trans, racial, or intersectional (race and anti-trans) discrimination impact the access to and sustainability of housing for trans women. By looking at factors associated with housing instability, we can identify factors leading to structural inequities. These data are being analyzed to inform efforts to address structural barriers to healthcare engagement among trans women in the San Francisco Bay Area.

## 2. Materials and Methods

We conducted a secondary analysis of data from the Trans *National study, which is an HIV incidence study of trans women in four cities around the world. Baseline data from the San Francisco Bay Area site was utilized for this analysis. Data was collected from 2016–2018. The study sample consisted of 629 trans women at least 18 years of age residing in the San Francisco Bay Area. The Trans *National study used respondent driven sampling (RDS), a method using social networks to best reach a diverse and hard to reach population, to recruit trans women [36]. The specific study procedures are discussed elsewhere [37]. Eligible participants were (1) over the age of 18, (2) identified on the trans-feminine spectrum, (3) lived in the San Francisco Bay Area, and (4) had not participated in the study before [38]. The study was approved by the Institutional Review Board at the University of California, San Francisco.

Anti-trans, racial, and intersectional (i.e., both racial and anti-trans) discrimination (the main independent variable) were assessed using a modified version of the Experiences of Discrimination (EOD) 10 item scale. The scale asks respondents if they had, “ever experienced discrimination, been prevented from doing something, or been hassled or made to feel inferior because of your gender identity/presentation, race/ethnicity/color, or both” in each of 10 situations (i.e., at work, at school, etc.) [39]. Response options were yes = 1, or no = 0. If participants reported yes, they were asked to clarify which form of discrimination they experienced (anti-trans, racial, or both). Items included whether they experienced discrimination while at work, looking for housing, on the street or in a public setting, at school, and while looking for employment, among others. Responses were summed across items to create a summary score for each discrimination type. For statistical analysis, each discrimination summary score was then made into an ordered categorical variable (0, 1–2, or 3 or more experiences) [39].

Housing status, the dependent variable, was assessed by asking respondents how they would describe their current living situation and attributed their responses to categories such as renting, couch surfing, staying with friends or family, owning a home, living in transitional or temporary housing, experiencing homelessness, or other. Housing status was then collapsed into a dichotomous variable (housing stability = 0 if owning or renting a home or staying in an SRO (Single Room Occupancy); housing instability = 1 if couch surfing, living in transitional or temporary housing, or experiencing homelessness).

**Figure 1:**
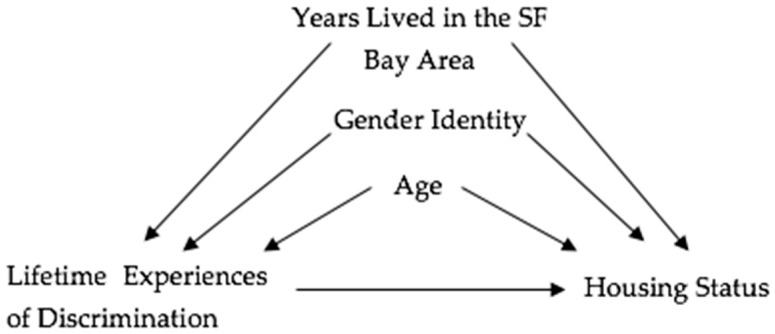
Directed Acyclic Graph (DAG) of hypothesized relationship among lifetime experiences of discrimination, housing status, age, gender identity, and years lived in the SF Bay Area.

We included independent covariates for gender identity, years lived in the Bay Area, and age based on past literature, our directed acyclic graph (DAG) (Figure 1) and because they may be associated with both reported lifetime discrimination and housing stability status [21,40,41,42]. Gender identity was dichotomized (gender identity 0 = self-identifying as female; gender identity 1 = gender identity other than sex assigned at birth). The amount of years lived in the Bay Area and age were both coded as continuous. Race/ethnicity was classified into five different categories: Black/African-American, White, Asian, Hispanic/Latina, and multiple races/ethnicities. Due to low frequency, multiple race/ethnicities and those who reported other were combined for analyses.

Descriptive statistics were calculated using univariate analysis for each type of discrimination, housing status, race/ethnicity, gender identity, age, and years lived in the Bay Area (Table 1). Cross tabulations were used to assess bivariate associations between race and ethnicity and types of discrimination. We included three multivariable logistic regression models, given our binary outcome of housing status (stability/instability), to analyze the association between anti-trans discrimination, racial discrimination, and intersectional discrimination and housing status adjusting for years lived in the Bay Area, age, and gender identity in the last 12 months (Table 2). We stratified by race and ethnicity to look at between-group differences for the three models (Table 3). Further detail of each EOD item was assessed to determine which item had the highest amount of anti-trans, racial, or intersectional discrimination. All analyses were performed using Stata/IC 15.0.

We ensured the logistic regression models met all of the assumptions and found no violations through a large enough sample size. We also ran a goodness-of-fit test, and found an ordered categorical variable for our exposure variable was the best fit. Data with missing values were excluded from the analysis (*n* = 5, 0.8%).

## 3. Results

### 3.1. Descriptive Statistics

The sample was racially and ethnically diverse; 32.6% (*n* = 205) identified as Hispanic/Latina, 17.0% (*n* = 107) identified as Black or African American, 28.9% (*n* = 182) identified as White, 18.3% (*n* = 115) identified as multiple ethnicities or other, and 3.2% (*n* = 20) identified as Asian (see Table 1**).** More than half of the study population reported their gender identity as a trans women or any other gender other than their sex assigned at birth (*n* = 345, 54.9%), while 45.2% identified as female. The median age of participants in the study was 40.05, and the median years of living in the Bay Area was 16.8 years.

Most trans women reported one to two (*n* = 225, 35.8%) or three or more (*n* = 273, 43.4%) lifetime experiences of anti-trans discrimination. Of those who reported anti-trans discrimination, the most reported item was when searching for housing at 21.4%. Relatively few trans women in the study reported racial discrimination alone (10.1%). The most reported form of racial discrimination experienced was “at school” (2.7%). Overall, more than half of all trans women in the study reported intersectional (racial and anti-trans) discrimination (*n* = 328, 52.2%). Of those who reported lifetime experiences of intersectional racial and anti-trans discrimination, the most reported item was while looking for housing (14.0%). Over a third of trans women in the study (*n* = 218, 34.7%) reported housing instability. In response to an open-ended question within the Trans *National study survey, housing instability was a primary concern voiced by trans women.

### 3.2. Statistical Analysis Results

Multivariable logistic regression results are shown in Table 2. Neither anti-trans discrimination (OR = 0.91, 95% CI = (0.73, 1.14)) nor racial discrimination alone (OR = 1.03, 95% CI= (0.63, 1.73)) were associated with housing instability. However, intersectional discrimination was associated with housing instability (OR = 1.25, 95% CI = (1.01, 1.54)) adjusting for years lived in the Bay Area, age, and trans identity. The adjusted odds of housing instability increased by 1.25 for every categorical unit increase (1–2, or 3+) in reported experiences of intersectional discrimination for trans women. Gender identity, length of stay in the San Francisco Bay Area, and age were not found to be confounders. 

The results did not show differences when stratifying by race/ethnicity (Table 3). When we stratified the results by race/ethnicity and analyzed the association between each form of discrimination and housing status by racial/ethnic group adjusting for years lived in the Bay Area, gender identity, and age, we found no significant associations among trans women.

## 4. Discussion

In our analysis, trans women who reported intersectional anti-trans and racial discrimination had a higher likelihood of experiencing housing instability. Our study is consistent with research showing discrimination is linked to housing instability for trans women but unique in our specific finding on the important influence of intersectional racial and anti-trans discrimination [13,35,41]. Prior studies have found race to have an association with housing instability. For example, a study found higher rates of housing instability among black trans women compared to their white trans peers [3,41]. Prior studies found housing instability to be associated with social stigmatization for being transgender, but we did not identify literature showing associations between housing instability and intersectional, or combined anti-trans and racial discrimination [8,13]. When our results were stratified by race and ethnicity, trans women in each specific racial/ethnic category did not have a higher risk of housing instability when they reported one or the other form of discrimination. Separating anti-trans and racial discrimination was commonly done in prior studies, which can inform interventions, but misses the intersectional experiences related to having more than one stigmatized social identity.

Using the concept of intersectionality requires we look at interlocking systems of oppression to better address the adverse health effects of those with multiple marginalized identities. The intersectionality framework, “asserts that race and gender constitute each other such that one identity alone (e.g., gender) cannot explain the unequal or disparate outcomes without the intersection of the other identity or identities” [43]. In a literature review documenting LGBTQ homelessness in the United States, it was found that intersectionality experienced by LGBTQ ethnic minorities led to a disproportionately higher risk of experiencing homelessness. This review found that LGBTQ ethnic minorities were at greater odds of experiencing discrimination, poverty, and victimization, which in turn led to housing instability and increased emotional distress and hardship [44]. With an added layer of racial discrimination, trans women who are racial/ethnic minorities have increased barriers to housing compared to their peers. Future research on housing among trans women would benefit from using an intersectionality framework in the approach to study design and analysis to identify multiple social categorizations as intertwined and impactful on multiple levels (systems, community, and interpersonal) [43,44,45]. As with all research, there are limitations to our study. Bias from unmeasured covariates that have been established as predictors of housing stability such as income, employment status, education, size and strength of social network, number of times moved in the Bay Area, or neighborhood may be present in our analysis and limit our models from being more comprehensive. Our data may not be generalizable to other trans populations as the housing crisis and trans health services are unique in San Francisco. The cross-sectional nature of the dataset also limits interpretations of causality. Specific questions that could have revealed further information related to housing and anti-trans discrimination in the initial survey were not included, such as when the instance of discrimination occurred in the housing process (i.e., from past evictions, or reported number of times denied housing). Despite limitations, we had a diverse large sample of trans women from many racial backgrounds as participants in the study, which allowed us to assess the impact of discrimination on housing stability and move the science forward on this important structural determinant of health.

### Implications

Policies and interventions focusing on both race and gender could eliminate intersectional (both anti-trans and racial) discrimination and structural barriers so that basic services such as housing can be accessed and downstream health outcomes (e.g., viral load suppression and adherence to ART) can be improved specifically for trans women. Research to examine the precursors to housing instability can inform anti-discrimination campaigns, which likely would impact a number of social determinants at the structural level that impact HIV risk and care outcomes, access to employment, food, and improved access to health care [12]. By creating policies and structural interventions, we can ensure trans women are not discriminated against when looking for housing or employment, create supportive housing for those rejected by family and others, and reduce poverty rates.

To eliminate barriers to care for trans individuals experiencing homelessness, the resources must be affirming and welcoming to trans women with intersecting identities by race and gender. Solutions include ensuring that the organizations fighting homelessness promote referrals to housing for trans women that are safe and culturally competent by race and gender [18]. Safe and culturally competent referral services for people with intersecting identities include services that respect gender identity, and that also have a historical understanding of transgender populations and the stigmatization often experienced in settings involving housing and employment, while also tailoring the services towards the client’s cultural background. This is different from services that are geared toward one’s race or for trans people alone because there is a mission to understand, respect, and address the multiple layers of a transgender woman’s identity.

Organizations supporting trans women must acknowledge the importance of intersectional identities and barriers to “wrap-around” services so they can advocate for trans women to access and stay retained in them, as these services focus on quality health care and housing as necessary components to improve health and quality of life. “Wrap-around” services (i.e., services that support for housing, employment, and health) that address intersecting identities are grounded in a holistic approach to help populations with multiple marginalized identities, to reduce health disparities among the most vulnerable in our society.

Further research is needed on how to eliminate structural barriers related to housing, income, and employment. Research focusing on practices in shelters becoming safer and welcoming for trans women, as well as on how intersectional racial and anti-trans discrimination is manifesting when they are looking for housing (i.e., discrimination of credit scores), is needed. This will help researchers better understand the mechanisms through which discrimination may lead to housing instability for trans women.

We may have found an association between intersectional discrimination and housing instability because supports for housing often do not focus on the multiple identities trans women have by race and gender. It may also be because intersectional racial and anti-trans discrimination impacts upon multiple portions of the lives of trans women. This may happen through rejection from family and other supports, and while they are also being discriminated against when looking for housing in San Francisco. This in turn impacts their quality of life where their health and safety are at risk from experiencing housing instability.

## 5. Conclusions

This study revealed that intersectional discrimination disproportionately burdens trans women and may increase the risk of experiencing housing instability. Our findings do not align with past research that has examined these factors within the lives of trans women, although it may be the first of its kind to examine intersectional discrimination as a risk factor that can lead to housing stability. While housing is an extremely dire issue in the San Francisco Bay Area, the added layers of intersectional discrimination are also major barriers for trans women seeking housing.

## Figures and Tables

**Table 1:** Demographic characteristics and experiences of anti-trans, racial and intersectional racial and anti-trans discrimination by trans women in the San Francisco Bay Area, 2016–2018.

Characteristics	*N* (%)
Independent Variables	
Gender Identity (1) Anti-Trans	284 (45.2)
Trans	345 (54.9)
Race/Ethnicity Hispanic/Latina Black/African-American White Asian Multiple/Other	205 (32.6) 107 (17.0) 182 (28.9) 20 (3.2) 115 (18.3)
Age Years Lived in SF Bay Area	Mean (40.1) Mean (16.8)
Main Exposure Anti-Trans Discrimination Racial Discrimination Intersectional (Race and Anti-Trans) Discrimination	498 (79.2) 64 (10.1) 328 (52.2)
Dependent/Outcome Variable	
Housing Stability Housing Instability	411 (65.3) 218 (34.7)

**Table 2:** Anti-trans, racial, or intersectional discrimination associations with housing instability among trans women in the San Francisco Bay Area.

Discrimination Type	Parameter	Coefficient Estimate	Standard Error	OR	95% CI	*p*-Value
*(1) Anti-Trans* log (p/1-p) = a + bx + cy + dw + ez log (p/1-p) = housing + b (anti-trans discrim) + c (gender) + d (age) + e (years in Bay Area)	a b c d e	0.57 −0.09 0.23 −0.03 −0.01	0.35 0.11 0.17 0.01 0.01	-- 0.91 1.25 0.98 0.99	-- (0.73, 1.14) (0.89, 1.77) (0.96, 0.99) (0.97, 0.99)	-- 0.42 0.20 0.01 * 0.04 *
*(2) Racial* log (p/1-p) = a + bx + cy + dw + ez log (p/1-p) = housing + b (racial discrim) + c (gender) + d (age) + e (years in Bay Area)	a b c d e	0.43 0.04 0.23 −0.03 −0.01	0.31 0.26 0.18 0.01 0.01	-- 1.03 1.25 0.98 0.99	-- (0.63, 1.73) (0.89, 1.77) (0.96, 0.99) (0.97, 0.99)	-- 0.88 0.19 0.01 * 0.05
*(3) Intersectional* log (p/1-p) = a + bx + cy + dw + ez log (p/1-p) = housing + b (intersectional discrim) + c (gender) + d (age) + e (years in Bay Area)	a b c d e	0.30 0.22 0.18 −0.03 −0.01	0.32 0.11 0.18 0.01 0.01	-- 1.25 1.19 0.98 0.98	-- (1.01, 1.54) (0.85, 1.70) (0.96, 0.99) (0.97, 0.99)	-- 0.03 * 0.31 0.01 * 0.05

All models adjust for gender identity, age, and years lived in the Bay Area. OR: Odds Ratio. CI: Confidence Interval. * *p* < 0.05.

**Table 3:** Stratifying by race/ethnicity, the odds of housing instability associated with exposure to anti-trans, racial, or intersectional discrimination among trans women in the San Francisco Bay Area, adjusting for gender identity, age, and years lived in the Bay Area.

**Discrimination Type**				**Hispanic/Latina**					**Multiple/Other**					**Black/African American**		
	**Parameter**	**Coeff.** **Est.**	**SE**	**OR**	**95% CI**	***p* Value**	**Coeff.** **Est.**	**SE**	**OR**	**95% CI**	***p* Value**	**Coeff.** **Est.**	**SE**	**OR**	**95% CI**	***p* Value**
*(4) Anti-trans* log (p/1-p) = a + bx + cy + dw + ez log (p/1-p) = housing + b (anti-trans discrim) + c (gender) + d (age) + e (years in Bay Area)	a b c d e	0.96 −0.17 0.69 −0.05 −0.01	0.62 0.20 0.33 0.02 0.01	-- 0.84 1.99 0.95 0.99	-- (0.56, 1.25) (1.05, 3.80) (0.93, 0.98) (0.96, 1.02)	-- 0.40 0.04 * 0.01 * 0.56	0.38 0.03 −0.56 0.01 −0.03	0.84 0.26 0.40 0.02 0.01	-- 1.03 0.57 1.00 0.97	-- (0.62, 1.72) (0.26, 1.26) (0.97, 1.04) (0.94, 0.99)	-- 0.91 0.17 0.90 0.03 *	1.33 0.26 0.21 −0.05 −0.01	0.87 0.30 0.45 0.02 0.01	-- 1.29 1.23 0.95 0.99	-- (0.73, 2.28) (0.51, 2.96) (0.92, 0.99) (0.97, 1.02)	-- 0.38 0.65 0.01 * 0.91
*(5) Racial* log (p/1-p) = a + bx + cy + dw + ez log (p/1-p) = housing + b (racial discrim) + c (gender) + d (age) + e (years in Bay Area)	a b c d e	0.75 −0.10 0.68 −0.05 −0.01	0.57 0.43 0.33 0.01 0.01	-- 0.90 1.96 0.96 0.99	-- (0.39, 2.08) (1.03, 3.74) (0.93, 0.98) (0.96, 1.02)	-- 0.81 0.04 * 0.01 * 0.62	0.46 −0.19 −0.55 0.01 −0.03	0.74 0.55 0.41 0.02 0.01	-- 0.82 0.58 1.00 0.97	-- (0.28, 2.40) (0.26, 1.28) (0.97, 1.04) (0.94, 0.99)	-- 0.72 0.18 0.93 0.03 *	1.49 0.26 0.26 −0.05 −0.01	0.85 0.52 0.45 0.02 0.01	-- 1.30 1.29 0.95 0.99	-- (0.47, 3.62) (0.54, 3.1) (0.92, 0.98) (0.97, 1.02)	-- 0.72 0.18 0.93 0.03 *
*(6) Intersectional* log (p/1-p) = a + bx + cy + dw + ez log (p/1-p) = housing + b (intersectional discrim) + c (gender) + d (age) + e (years in Bay Area)	a b c d e	0.68 0.08 0.67 −0.05 −0.01	0.58 0.19 0.33 0.15 0.01	-- 1.09 1.96 0.95 0.99	-- (0.75, 1.59) (1.03, 3.73) (0.93, 0.98) (0.97, 1.02)	-- 0.64 0.04 * 0.01 * 0.59	0.22 0.20 −0.62 0.01 −0.03	0.77 0.25 0.41 0.02 0.01	-- 1.22 0.54 1.00 0.97	-- (0.75, 2.00) (0.24, 1.21) (0.97, 1.04) (0.94, 0.99)	-- 0.42 0.14 0.83 0.03 *	1.00 0.41 0.21 −0.05 0.01	0.92 0.27 0.45 0.02 0.01	-- 1.50 1.24 0.95 1.00	-- (0.89, 2.53) (0.51, 2.99) (0.92, 0.99) (0.98, 1.03)	-- 0.13 0.64 0.01 * 0.78
**Discrimination Type**						**White**								**Asian**		
	**Parameter**		**Coeff.** **Est.**	**SE**		**OR**	**95% CI**	***p* Value**		**Coeff.** **Est.**		**SE**		**OR**	**95% CI**	***p* Value**
*(4) Anti-trans* log (p/1-p) = a + bx + cy + dw + ez log (p/1-p) = housing + b (anti-trans discrim) + c (gender) + d (age) + e (years in Bay Area)	a b c d e		−0.53 0.23 −0.06 −0.01 −0.05	0.79 0.32 0.37 0.02 0.02		-- 1.25 0.95 0.99 0.95	-- (0.68, 2.34) (0.46, 1.94) (0.96, 1.02) (0.92, 0.99)	-- 0.46 0.88 0.69 0.01 *		0.25 0.43 2.02 −0.08 0.03		2.29 0.73 1.66 0.09 0.05		-- 1.54 7.56 0.92 1.03	-- (0.37, 6.46) (0.29, 194) (0.77, 1.09) (0.94, 1.12)	-- 0.55 0.22 0.36 0.59
*(5) Racial* log (p/1-p) = a + bx + cy + dw + ez log (p/1-p) = housing + b(racial discrim) + c (gender) + d (age) + e (years in Bay Area)	a b c d e		−0.15 0.21 −0.02 −0.01 −0.05	1.26 0.36 0.02 0.02 0.59		-- 1.23 0.98 0.99 0.95	-- (0.10, 14.7) (0.48, 1.99) (0.96, 1.02) (0.92, 0.99)	-- 0.87 0.96 0.66 0.01 *		1.77 0 3.01 −0.12 0.03		2.54 0 1.87 0.09 0.04		-- 1 20.3 0.88 1.03	-- -- (0.52, 796) (0.73, 1.07) (0.94, 1.14)	-- -- 0.12 0.22 0.49
*(6) Intersectional* log (p/1-p) = a + bx + cy + dw + ez log (p/1-p) = housing + b (intersectional discrim) + c (gender) + d (age) + e (years in Bay Area)	a b c d e		−0.16 0.34 −0.01 −0.01 −0.05	0.59 0.31 0.36 0.02 0.02		-- 1.41 0.99 0.99 0.95	-- (0.76, 2.6) (0.48, 2.02) (0.96, 1.02) (0.92, 0.99)	-- 0.27 0.98 0.59 0.01 *		−0.01 0.14 1.58 −0.06 0.03		2.43 0.61 1.45 0.08 0.05		-- 1.15 4.86 0.94 1.03	-- (0.35, 3.83) (0.28, 83.5) (0.80, 1.09) (0.94,1.12)	-- 0.82 0.28 0.44 0.59

All models adjust for gender identity, age, and years lived in the Bay Area. OR: Odds Ratio. CI: Confidence Interval. * *p* < 0.05.
